# Open-ended interview questions and saturation

**DOI:** 10.1371/journal.pone.0198606

**Published:** 2018-06-20

**Authors:** Susan C. Weller, Ben Vickers, H. Russell Bernard, Alyssa M. Blackburn, Stephen Borgatti, Clarence C. Gravlee, Jeffrey C. Johnson

**Affiliations:** 1 Department of Preventive Medicine & Community Health, University of Texas Medical Branch, Galveston, Texas, United States of America; 2 Institute for Social Research, Arizona State University, Tempe, Arizona/University of Florida, Gainesville, Florida, United States of America; 3 Department of Molecular and Human Genetics, Baylor College of Medicine, Houston, Texas, United States of America; 4 Department of Management, University of Kentucky, Lexington, Kentucky, United States of America; 5 Department of Anthropology, University of Florida, Gainesville, Florida, United States of America; University of Birmingham, UNITED KINGDOM

## Abstract

Sample size determination for open-ended questions or qualitative interviews relies primarily on custom and finding the point where little new information is obtained (thematic saturation). Here, we propose and test a refined definition of saturation as obtaining *the most salient items* in a set of qualitative interviews (where items can be material things or concepts, depending on the topic of study) rather than attempting to obtain *all the items*. Salient items have higher prevalence and are more culturally important. To do this, we explore saturation, salience, sample size, and domain size in 28 sets of interviews in which respondents were asked to list all the things they could think of in one of 18 topical domains. The domains—like kinds of fruits (highly bounded) and things that mothers do (unbounded)—varied greatly in size. The datasets comprise 20–99 interviews each (1,147 total interviews). When saturation was defined as the point where less than one new item per person would be expected, the median sample size for reaching saturation was 75 (range = 15–194). Thematic saturation was, as expected, related to domain size. It was also related to the amount of information contributed by each respondent but, unexpectedly, was reached more quickly when respondents contributed *less* information. In contrast, a greater amount of information per person increased the retrieval of *salient* items. Even small samples (*n* = 10) produced 95% of the most salient ideas with exhaustive listing, but only 53% of those items were captured with limited responses per person (three). For most domains, item salience appeared to be a more useful concept for thinking about sample size adequacy than finding the point of thematic saturation. Thus, we advance the concept *of saturation in salience* and emphasize probing to increase the amount of information collected per respondent to increase sample efficiency.

## Introduction

Open-ended questions are used alone or in combination with other interviewing techniques to explore topics in depth, to understand processes, and to identify potential causes of observed correlations. Open-ended questions may produce lists, short answers, or lengthy narratives, but in all cases, an enduring question is: How many interviews are needed to be sure that the range of salient *items* (in the case of lists) and *themes* (in the case of narratives) are covered. Guidelines for collecting lists, short answers, and narratives often recommend continuing interviews until *saturation* is reached. The concept of *theoretical saturation*—the point where the main ideas and variations relevant to the formulation of a theory have been identified—was first articulated by Glaser and Strauss [1,2] in the context of how to develop grounded theory. Most of the literature on analyzing qualitative data, however, deals with observable *thematic saturation*—the point during a series of interviews where few or no new ideas, themes, or codes appear [3–6].

Since the goal of research based on qualitative data is not necessarily to collect all or most ideas and themes but to collect the most important ideas and themes, salience may provide a better guide to sample size adequacy than saturation. Salience (often called cultural or cognitive salience) can be measured by the frequency of item occurrence (prevalence) or the order of mention [7,8]. These two indicators tend to be correlated [9]. In a set of lists of birds, for example, robins are reported more frequently and appear earlier in responses than are penguins. Salient terms are also more prevalent in everyday language [10–12]. Item salience also may be estimated by combining an item’s frequency across lists with its rank/position on individual lists [13–16].

In this article, we estimate the point of complete thematic saturation and the associated sample size and domain size for 28 sets of interviews in which respondents were asked to list all the things they could think of in one of 18 topical domains. The domains—like kinds of fruits (highly bounded) and things that mothers do (unbounded)—varied greatly in size. We also examine the impact of the amount of information produced per respondent on saturation and on the number of unique items obtained by comparing results generated by asking respondents to name *all* the relevant things they can with results obtained from a limited number of responses per question, as with standard open-ended questioning. Finally, we introduce an additional type of saturation based on the relative salience of items and themes—*saturation in salience*—and we explore whether the most salient items are captured at minimal sample sizes. *A key conclusion is that saturation may be more meaningfully and more productively conceived of as the point where the most salient ideas have been obtained*.

### Recent research on saturation

Increasingly, researchers are applying systematic analysis and sampling theory to untangle the problems of saturation and sample size in the enormous variety of studies that rely on qualitative data—including life-histories, discourse analysis, ethnographic decision modeling, focus groups, grounded theory, and more. For example, Guest et al.[17] and others[18–19] found that about 12–16 interviews were adequate to achieve thematic saturation. Similarly, Hagaman and Wutich [20] found that they could reliably retrieve the three most salient themes from each of the four sites in the first 16 interviews.

Galvin[21] and Fugard and Potts[22] framed the sample size problem for qualitative data in terms of the likelihood that a specific idea or theme will or will not appear in a set of interviews, given the prevalence of those ideas in the population. They used traditional statistical theory to show that small samples retrieve only the most prevalent themes and that larger samples are more sensitive and can retrieve less prevalent themes as well. This framework can be applied to the expectation of observing or not observing almost anything. Here it would apply to the likelihood of observing a theme in a set of narrative responses, but it applies equally well for situations such as behavioral observations, where specific behaviors are being observed and sampled[23]. For example, to obtain ideas or themes that would be reported by about one out of five people (0.20 prevalence) or a behavior with the same prevalence, there is a 95% likelihood of seeing those themes or behaviors *at least once* in 14 interviews—if those themes or behaviors are independent.

Saturation and sample size have also begun to be examined with multivariate models and simulations. Tran et al. [24] estimated thematic saturation and the total number of themes from open-ended questions in a large survey and then simulated data to test predictions about sample size and saturation. They assumed that items were independent and found that sample sizes greater than 50 would add less than one new theme per additional person interviewed.

Similarly, Lowe et al. [25] estimated saturation and domain size in two examples and in simulated datasets, testing the effect of various parameters. Lowe et al. found that responses were not independent across respondents and that saturation may never be reached. In this context, non-independence refers to the fact that some responses are much more likely than others to be repeated across people. Instead of complete saturation, they suggested using a goal such as obtaining a percentage of the total domain that one would like to capture (e.g., 90%) and the average prevalence of items one would like to observe to estimate the appropriate sample size. For example, to obtain 90% of items with an average prevalence of 0.20, a sample size of 36 would be required. Van Rijnsoever [26] used simulated datasets to study the accumulation of themes across sample size increments and assessed the effect of different sampling strategies, item prevalence, and domain size on saturation. Van Rijnsoever’s results indicated that the point of saturation was dependent on the prevalence of the items.

As modeling estimates to date have been based on only one or two real-world examples, it is clear that more empirical examples are needed. Here, we use 28 real-world examples to estimate the impact of sample size, domain size, and amount of information per respondent on saturation and on the total number of items obtained. Using the proportion of people in a sample that mentioned an item as a measure of salience, we find that even small samples may adequately capture the most salient items.

## Materials and methods

### The data

The datasets comprise 20–99 interviews each (1,147 total interviews). Each example elicits multiple responses from each individual in response to an open-ended question (“Name all the … you can think of”) or a question with probes (“What other … are there?”).

Data were obtained by contacting researchers who published analyses of free lists. Examples with 20 or more interviews were selected so that saturation could be examined incrementally through a range of sample sizes. Thirteen published examples were obtained on: illness terms [27] (in English and in Spanish); birds, flowers, and fabrics [28]; recreational/street drugs and fruits [29]; things mothers do (online, face-to-face, and written administration) and racial and ethnic groups [30] (online, face-to-face, and written administration). Fifteen unpublished classroom educational examples were obtained on: soda pops (Weller, n.d.); holidays (two replications), things that might appear in a living room, characteristics of a good leader (two replications), a good team (two replications), and a good team player (Johnson, n.d.); and bad words, industries (two replications), cultural industries (two replications), and scary things (Borgatti, n.d.). (Original data appear online in S1 Appendix The Original Data for the 28 Examples.)

Some interviews were face to face, some were written responses, and some were administered on-line. Investigators varied in their use of prompts, using nonspecific (What other … are there?), semantic (repeating prior responses and then asking for others), and/or alphabetic prompts (going through the alphabet and asking for others). Brewer [29] and Gravlee et al. [30] specifically examined the effect of prompting on response productivity, although the Brewer et al. examples in these analyses contain results before extensive prompting and the Gravlee et al. examples contain results after prompting. The 28 examples, their topic, source, sample size, the question used in the original data collection, and the three most frequently mentioned items appear in Table 1. All data were collected and analyzed without personal identifying information.

**Table 1:** The examples.

DOMAIN	INTERVIEW MODE (SAMPLE SIZE)	QUESTION ASKED	THE MOST FREQUENTLY MENTIONED ITEMS (Prevalence)
1. Fruits (Brewer et al. 2002)	oral (*n* = 33)	“Think of all the different kinds of fruit people eat. Tell me the names of all the kinds of fruit you can remember. Please keep trying to recall if you think there are more kinds of fruit you might be able to remember.”	#1/Apple (0.97), #47/Orange (0.97), #4/Banana (0.91)
2. Birds (Brewer 1995)	written (*n* = 36)	“What are all the kinds of birds? Please write down the names of all the birds you can think of.” [in 10 minutes]	Eagle (0.83), Crow (0.83), Hummingbird (0.81).
3. Flowers (Brewer 1995)	written (*n* = 41)	“What are all the kinds of flowers? Please write down the names of all the flowers you can think of.” [in 10 minutes]	Rose (1.0), Carnation (0.98), Tulip (0.80), Daisy (0.80).
4. Drugs (Brewer et al. 2002)	oral (*n* = 43, drug injectors)	“Think of all the different kinds of drugs or substances people use to get high, feel good, or think and feel differently. These drugs are sometimes called recreational drugs or street drugs. Tell me the names of all the kinds of these drugs you can remember. Please keep trying to recall if you think there are more kinds of drugs you might be able to remember.	#50/Heroin (0.93), #18/Cocaine (0.93), #59/Marijuana (0.93).
5. Fabrics (Brewer 1995)	(*n* = 63)	“What are all the kinds of fabrics? Please write down the names of all the fabrics you can think of.” [in 10 minutes]	Cotton (1.0), Polyester (0.98), Silk (0.94), Wool (0.94)
6. Illnesses-US (Weller 1983)	face-to-face (*n* = 20)	“Tell me all the illnesses you know of.” [Nonspecific and semantic prompts]	Cancer (0.75), Measles (0.65), Mumps (0.65).
7. Illnesses-G (Guatemala) (Weller 1983)	face-to-face (*n* = 20)	“Puede Ud. decirme todas las enfermedades que conozca o que recuerde, por favor?” [Nonspecific and semantic prompts]	Sarampion (0.75), Varicela (0.60), Gripe (0.55), Amigdalitis (0.55)
8. Sodas (Weller, n.d.)	written (*n* = 28)	“Please write down all the names for sodas or soda pops that you can think of. You have 3 minutes.”	Coca Cola (1.0), Pepsi (0.96), and Sprite (0.93).
9. Holiday1 (Johnson, n.d.)	written (*n* = 24)	“Write down all the holidays you can think of.”	Christmas (0.88), Memorial Day (0.83), July 4th (0.83).
10. Holiday2 (Johnson, n.d.)	written (*n* = 23)	“Write down all the holidays you can think of.”	Christmas (1.0), Memorial Day (1.0), Thanksgiving (0.96).
11. LivingRoom (Johnson, n.d.)	written (*n* = 33)	“List all the things you would find in an American living room.”	Couch (0.91), TV (0.88), Coffee table (0.76)
12. GoodLeader (Johnson, n.d.)	written (*n* = 36)	“What are the characteristics of a good team leader?” [in 5 min]	Good listener (0.33), Communicator (0.28), Good example (0.22)
13. GoodTLeader (Johnson, n.d.)	written (*n* = 31)	“What are the characteristics of a good team leader?” [in 5 min]	Listener (0.45), Decisive (0.39), Honest (0.29), Respects others (0.29)
14. GoodTeam1 (Johnson, n.d.)	written (*n* = 36)	“What are the characteristics of a good team?	Goals (0.50), Cooperation working together (0.44), Respect (0.31)
15. GoodTeam3 (Johnson, n.d.)	written (*n* = 31)	“What are the characteristics of a good team?	Goals (0.45), Communication (0.39), Open-communication (0.39)
16. GoodTeam2 Player (Johnson, n.d.)	written (*n* = 36)	“What are the characteristics of a good team player?”	Cooperative (0.31), Understands role (0.28), Respectful (0.22), Listens (0.22)
17. Bad words (Borgatti, n.d.)	written (*n* = 92)	“What are all the bad words you can think of?”	Shit (0.90), Fuck (0.85), Asshole (0.73), Bitch (0.73).
18. Industries1 (Borgatti, n.d.)	written (*n* = 27)	“What are all the industries you can think of?”	Automobile (0.70), Construction (0.67), Banking (0.63), Entertainment (0.63).
19. Industries2 (Borgatti, n.d.)	written (*n* = 43)	“List all of the industries you can think of.”	Automobile (0.77), Health (0.63), Banking (0.60).
20. CultInd1 (Borgatti, n.d.)	written (*n* = 44)	“Please list all of the cultural industries you can think of.”	Museum (0.39), Television (0.34), Music (0.30).
21. CultInd2 (Borgatti, n.d.)	written (*n* = 29)	“Please list all of the cultural industries you can think of.”	Museum (0.34), School (0.31), Dance (0.24).
22. ScaryThings (Borgatti, n.d.)	written (*n* = 99)	“List all the scary things you can think of.”	Death (0.79), Heights (0.62), The dark (0.61).
23. Moms-OL (Gravlee et al., 2013)	online (*n* = 56) *n* = 55?	“We’re studying things that mothers do, so, with this in mind, please start by listing all the things that mothers do. There’s no limit and there are no right or wrong answers, so take your time.” [Semantic prompting]	Cooking (0.56), Loving (0.51), Household Cleaning (0.44).
24. Moms-F2F (Gravlee et al., 2013)	face-to-face (*n* = 50)	“Please list the all the things that mothers do. List as many things as you can think of. There’s no limit, so take your time.” [Semantic prompting]	Cooking (0.74), Working (0.58), Household Cleaning (0.58).
25. Moms-PP (Gravlee et al. 2013)	paper and pen (*n* = 53)	“Please list the things that mothers do. List as many things as you can think of. There’s no limit, so take your time. Write each thing one below the other.” [Semantic prompting]	Cooking (0.74), Loving (0.60), Household Cleaning (0.53).
26. Ethnic-OL (Gravlee et al. 2013)	online (*n* = 56)	“We’re studying the names of racial and ethnic groups, so, with this in mind, please start by listing all the racial and ethnic groups you can think of. There’s no limit and there are no right or wrong answers, so take your time.” [Semantic prompting]	White (0.79), Hispanic (0.68), Indian (0.64).
27. Ethnic-F2F (Gravlee et al. 2013)	face-to-face (*n* = 48)	“Please list as many racial and ethnic groups as you can think of. There’s no limit, so take your time. [Semantic prompting]	Chinese (0.88), Asian (0.88), Japanese (0.85).
28. Ethnic-PP (Gravlee et al. 2013)	paper and pen (*n* = 53)	“Please list as many racial and ethnic groups as you can think of. There’s no limit, so take your time. Write each group one below the other.” [Semantic prompting]	White (0.83), Hispanic (0.68), Asian (0.66).

### Analysis

For each example, statistical models describe the pattern of obtaining new or unique items with incremental increases in sample size. Individual lists were first analyzed with Flame [31,32] to provide the list of unique items for each example and the Smith [14] and Sutrop [15] item salience scores. Duplicate items due to spelling, case errors, spacing, or variations were combined.

To help develop an interviewing stopping rule, a simple model was used to predict the unique number of items contributed by each additional respondent. Generalized linear models (GLM, log-linear models for count data) were used to predict the unique number of items added by each respondent (incrementing sample size), because number of unique items added by each respondent (count data) is approximately Poisson distributed. For each example, models were fit with ordinary least squares linear regression, Poisson, and negative binomial probability distributions. Respondents were assumed to be in random order, in the order in which they occurred in each dataset, although in some cases they were in the order they were interviewed. Goodness-of-fit was compared across the three models with minimized deviants (the Akaike Information Criterion, AIC) to find the best-fitting model [33]. Using the best-fitting model for each example, the point of saturation was estimated as the point where the expected number of new items was one or less. Sample size and domain size were estimated at the point of saturation, and total domain size was estimated for an infinite sample size from the model for each example as the limit of a geometric series (assuming a negative slope).

Because the GLM models above used only incremental sample size to predict the total number of unique items (domain size) and ignored variation in the number of items provided by each person and variation in item salience, an additional analysis was used to estimate domain size while accounting for subject and item heterogeneity. For that analysis, domain size was estimated with a capture-recapture estimation technique used for estimating the size of hidden populations. Domain size was estimated from the total number of items on individual lists and the number of matching items between pairs of lists with a log-linear analysis. For example, population size can be estimated from the responses of two people as the product of their number of responses divided by the number of matching items (assumed to be due to chance). If Person#1 named 15 illness terms and Person#2 named 31 terms and they matched on five illnesses, there would be 41 unique illness terms and the estimated total number of illness terms based on these two people would be (15 x 31) /5 = 93.

A log-linear solution generalizes this logic from a 2 x 2 table to a 2^K^ table [34]. the capture–recapture solution estimates total population size for hidden populations using the pattern of recapture (matching) between pairs of samples (respondents) to estimate the population size. An implementation in R with GLM uses a log-linear form to estimate population size based on recapture rates (Rcapture [35,36]). In this application, it is assumed that the population does not change between interviews (closed population) and models are fit with: (1) no variation across people or items (M_*0*_); (2) variation only across respondents (M_*t*_); (3) variation only across items (M_*h*_); and (4) variation due to an interaction between people and items (M_*ht*_). For each model, estimates were fit with binomial, Chao’s lower bound estimate, Poisson, Darroch log normal, and gamma distributions [35]. Variation among items (heterogeneity) is a test for a difference in the probabilities of item occurrence and, in this case, is equivalent to a test for a difference in item salience among the items. Due to the large number of combinations needed to estimate these models, Rcapture software estimates are provided for all four models only up to a sample of size 10. For larger sample sizes (all examples in this study had sample sizes of 20 or larger), only model 1 with no effects for people or items (the binomial model) and model 3 with item effects (item salience differences) were tested. Therefore, models were fit at size 10, to test all four models and then at the total available sample size.

## Results

Descriptive information for the examples appears in Table 2. The first four columns list the name of the example, the sample size in the original study, the mean list length (with the range of the list length across respondents), and the total number of unique items obtained. For the Holiday1 example, interviews requested names of holidays (“Write down all the holidays you can think of”), there were 24 respondents, the average number of holidays listed per person (list length) was 13 (ranging from five to 29), and 62 unique holidays were obtained.

**Table 2:** Estimated point of saturation and domain size.

Example	*N*	Mean # Responses (range)	Total Unique Items	N_SAT_ (Y<1.0)	Domain Size at Y<1.0 (D_SAT_)	Est. Total Domain Size (D_TOT_)	Capture-Recapture Pop Size
Fruits	33	22.1 (12–43)	62	15	48	53	73^c^
Birds	36	25.9 (10–41)	121	35	114	130	203^c^
Flowers	41	16.1 (7–42)	141	33	130	143	251^c^
Drugs	43	13.6 (3–33)	92	33	84	101	115^c^
Fabrics	63	15.0 (5–28)	143	75	155	210	753^c^
Illnesses-US	20	16.1 (6–31)	144	53	217	237	758^g^
Illnesses-G	20	12.3 (4–28)	86	21	82	90	281^g^
Sodas	28	16.3 (7–27)	108	42	127	147	210^c^
Holiday1	24	13.0 (5–29)	62	17	48	57	100^c^
Holiday2	23	17.8 (8–39)	90	87	209	263	200^c^
LivingRoom	33	12.9 (6–24)	107	41	115	137	210^c^
GoodLeader	36	6.6 (3–15)	151	78	221	261	1165^g^
GoodTLeader	31	9.7 (3–19)	141	102	280	336	619^g^
GoodTeam1	36	6.4 (2–13)	136	96^p^	239	297	1615^g^
GoodTeam3	31	9.0 (1–18)	135	88	240	289	607^g^
GoodTeam2 Player	36	5.8 (2–14)	136	189^p^	428	555	1403^g^
BadWords	92	15.8 (3–35)	273	119	302	372	867^c^
Industries1	27	34.5 (7–69)	413	138	875	919	1189^c^
Industries2	43	34.9 (7–67)	510	194	1039	1108	1089^c^
CultInd1	44	12.2 (3–30)	299	50^nbi^	308	310	2224^g^
CultInd2	29	9.7 (1–26)	203	50	239	256	2478^g^
ScaryThings	99	16.9 (3–36)	453	177	577	662	1213^c^
MomsOL	55	17.8 (3–53)	389	104	477	516	881^c^
MomsF2F	50	30.2 (9–75)	560	161	896	951	1631^c^
MomsP&P	53	16.6 (5–51)	337	109	443	488	738^c^
EthnicOL	56	24.6 (5–90)	304	70	312	338	615^c^
EthnicF2F	48	33.3 (12–106)	339	91	415	449	684^c^
EthnicPP	53	17.9 (3–62)	228	58	234	257	521^c^

nbi = Negative binomial-identity, p = Poisson-log ; c = Chao’s Lower bound; g = gamma

### Predicting thematic saturation from sample size

The free-list counts showed a characteristic *descending curve* where an initial person listed new themes and each additional person repeated some themes already reported and added new items, but fewer and fewer new items were added with incremental increases in sample size. All examples were fit using the GLM log-link and identity-link with normal, Poisson, and negative binomial distributions. The negative binomial model resulted in a better fit than the Poisson (or identity-link models) for most full-listing examples, providing the best fit to the downward sloping curve with a long tail. Of the 28 examples, only three were not best fit by negative binomial log-link models: the best-fitting model for two examples was the Poisson log-link model (GoodTeam1 and GoodTeam2Player) and one was best fit by the negative binomial identity-link model (CultInd1).

Sample size was a significant predictor of the number of new items for 21 of the 28 examples. Seven examples did not result in a statistically significant fit (Illnesses-US, Holiday2, Industries1, Industries2, GoodTLeader, GoodTeam2Player, and GoodTeam3). The best-fitting model was used to predict the point of saturation and domain size for all 28 examples (S2 Appendix GLM Statistical Model Results for the 28 Examples).

Using the best-fitting GLM models we estimated the predicted sample size for reaching saturation. Saturation was defined as the point where less than one new item would be expected for each additional person interviewed. Using the models to solve for the sample size (X) when only one item was obtained per person (Y = 1) and rounding up to the nearest integer, provided the point of saturation (Y≤1.0). Table 2, column five, reports the sample size where saturation was reached (N_SAT_). For Holiday1, one or fewer new items were obtained per person when X = 16.98. Rounding up to the next integer provides the saturation point (N_SAT_ = 17). For the Fruit domain, saturation occurred at a sample size of 15.

Saturation was reached at sample sizes of 15–194, with a median sample size of 75. Only five examples (Holiday1, Fruits, Birds, Flowers, and Drugs) reached saturation within the original study sample size and most examples did not reach saturation even after four or five dozen interviews. A more liberal definition of saturation, defined as the point where less than two new items would be expected for each additional person (solving for Y≤2), resulted in a median sample size for reaching saturation of 50 (range 10–146).

**Fig 1:**
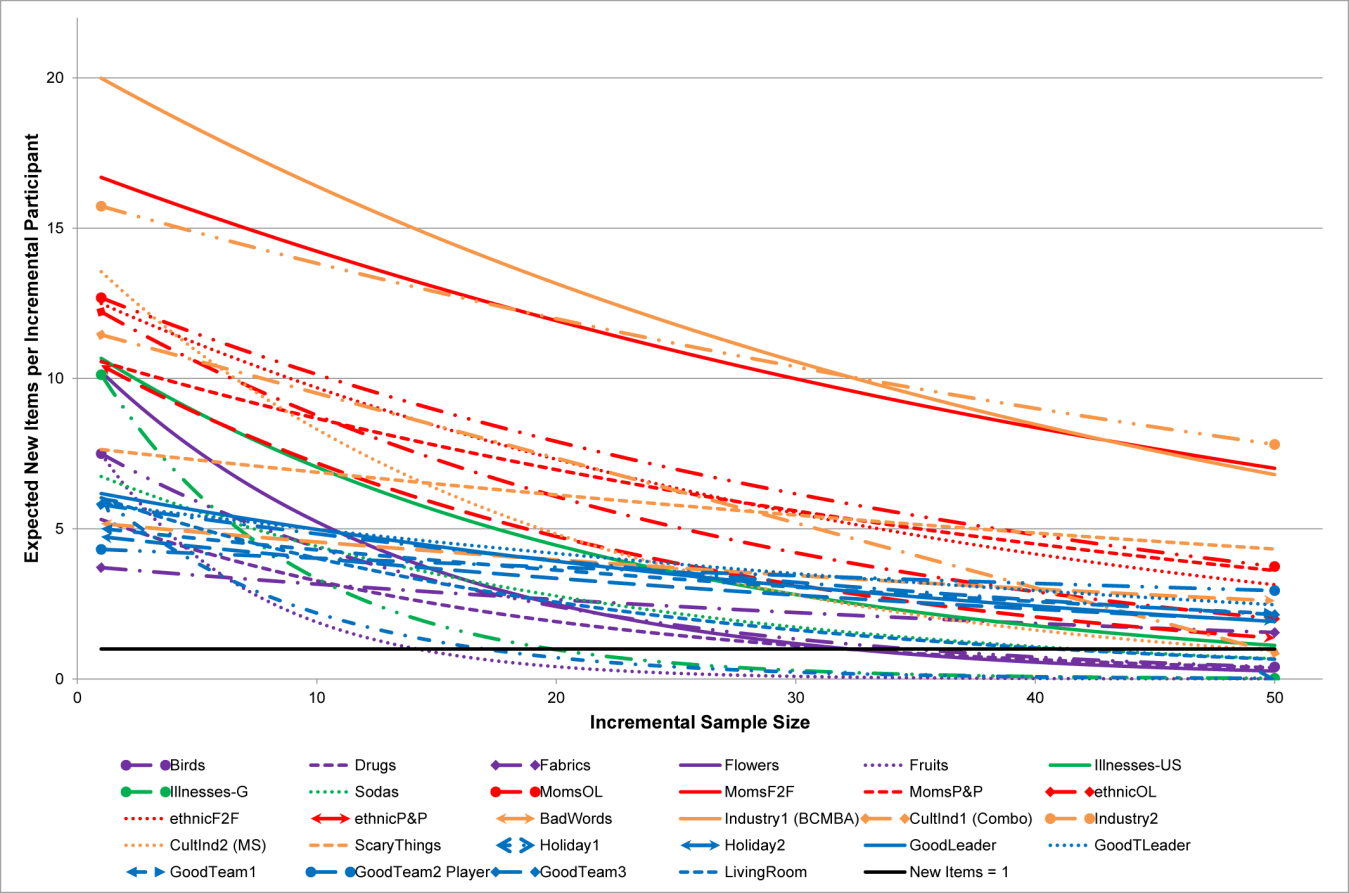
The number of unique items provided with increasing sample size.

Some domains were well bounded and were elicited with small sample sizes. Some were not. In fact, most of the distributions exhibited a very long tail—where many items were mentioned by only one or two people. Fig 1 shows the predicted curves for all examples for sample sizes of 1 to 50. Saturation is the point where the descending curve crosses Y = 1 (or Y = 2). Although the expected number of unique ideas or themes obtained for successive respondents tends to decrease as the sample size increases, this occurs rapidly in some domains and slowly or not at all in other domains. Fruits, Holiday1, and Illness-G are domains with the three bottom-most curves and the steepest descent, indicating that saturation was reached rapidly and with small sample sizes. The three top-most curves are the Moms-F2F, Industries1, and Industries2 domains, which reached saturation at very large sample sizes or essentially did not reach saturation.

### Estimating domain size

Because saturation appeared to be related to domain size and some investigators state that a percentage of the domain might be a better standard [25], domain size was also estimated. First, total domain size was estimated with the GLM models obtained above. Domain size was estimated at the point of saturation by cumulatively summing the number of items obtained for sample sizes *n* = 1, *n* = 2, *n* = 3, … to N_SAT_. For the Holiday1 sample, summing the number of predicted unique items for sample sizes *n* = 1 to *n* = 17 should yield 51 items (Table 2, Domain Size at Saturation, D_SAT_). Thus, the model predicted that approximately 51 holidays would be obtained by the time saturation was reached.

The total domain size was estimated using a geometric series, summing the estimated number of unique items obtained cumulatively across people in an infinitely large sample. For the Holiday1 domain, the total domain size was estimated as 56 (see Table 2, Total Domain Size D_TOT_). So for the Holiday1 domain, although the total domain size was estimated to be 57, the model predicted that saturation occurred when the sample size reached 17, and at that point 51 holidays should be retrieved. Model predictions were close to the empirical data, as 62 holidays were obtained with a sample of 24.

Larger sample sizes were needed to reach saturation in larger domains; the largest domains were MomsF2F, Industries1, and Industries2 each estimated to have about 1,000 items and more than 100 interviews needed to approach saturation. Saturation (Y≤1) tended to occur at about 90% of the total domain size. For Fruits, the domain size at saturation was 51 and the total domain size was estimated at 53 (51/53 = 96%) and for MomsF2F, domain size at saturation was 904 and total domain size was 951 (95%).

Second, total domain size was estimated using a capture-recapture log-linear model with a parameter for item heterogeneity [35,36]. A descending, concave curve is diagnostic of item heterogeneity and was present in almost all of the examples. The estimated population sizes using R-Capture appear in the last column of Table 2. When the gamma distribution provided the best fit to the response data, the domain size increased by an order of magnitude as did the standard error on that estimate. When responses fit a gamma distribution, the domain may be extremely large and may not readily reach saturation.

Inclusion of the pattern of matching items across people with a parameter for item heterogeneity (overlap in items between people due to salience) resulted in larger population size estimates than those above without heterogeneity. Estimation from the first two respondents was not helpful and provided estimates much lower than those from any of the other methods. The simple model without subject or item effects (the binomial model) did not fit any of the examples. Estimation from the first 10 respondents in each example suggested that more variation was due to item heterogeneity than to item and subject heterogeneity, so we report only the estimated domain size with the complete samples accounting for item heterogeneity in salience.

Overall, the capture–recapture estimates incorporating the effect of salience were larger than the GLM results above without a parameter for salience. For Fruits, the total domain size was estimated as 45 from the first two people; as 88 (gamma distribution estimate) from the first 10 people with item heterogeneity and as 67 (Chao lower bound estimate) with item and subject heterogeneity; and using the total sample (*n* = 33) the binomial model (without any heterogeneity parameters) estimated the domain size as 62 (but did not fit the data) and with item heterogeneity the domain size was estimated as 73 (the best-fitting model used the Chao lower bound estimate). Thus, the total domain size for Fruits estimated with a simple GLM model was 53 and with a capture–recapture model (including item heterogeneity) was 73 (Table 2, last column). Similarly, the domain size for Holiday1 was estimated at 57 with the simple GLM model and 100 with capture-recapture model. Domain size estimates suggest that even the simplest domains can be large and that inclusion of item heterogeneity increases domain size estimates.

### Saturation and the number of responses per person

The original examples used an exhaustive listing of responses to obtain about a half dozen (GoodLeader and GoodTeam2Player) to almost three dozen responses per person (Industries1 and Industries2). A question is whether saturation and the number of unique ideas obtained might be affected by the number of responses per person. Since open-ended questions may obtain only a few responses, we limited the responses to a maximum of three per person, truncating lists to see the effect on the number of items obtained at different sample sizes and the point of saturation.

When more information (a greater number of responses) was collected per person, more unique items were obtained even at smaller sample sizes (Table 3). The amount of information retrieved per sample can be conceived of in terms of bits of information per sample and is roughly the average number of responses per person times the sample size so that, with all other things being equal, larger sample sizes with less probing should approach the same amount of information obtained with smaller samples and more probing. So, for a given sample size, a study with six responses per person should obtain twice as much information as a study with three responses per person. In the GoodLeader, GoodTeam1, and GoodTeam2Player examples, the average list length was approximately six and when the sample size was 10 (6 x 10 = 60 bits of information), approximately twice as many items were obtained as when lists were truncated to three responses (3 x 10 = 30 bits of information).

**Table 3:** Comparison of number of unique items obtained with full free lists and with three or fewer responses.

Example	*N*	Mean # Responses	Total Unique Items Obtained	Free-list Unique Items		Three-list Unique Items		Three- list NSat (Y<1.0)
				*n* = 10	*n* = 20	*n* = 10	*n* = 20	
Fruits	33	22.1	62	51	59	15	15	9
Birds	36	25.9	121	85	92	21	28	15
Flowers	41	16.1	141	92	113	15	21	11
Drugs	43	13.6	92	42	65	11	16	8
Fabrics	63	15	143	52	71	12	16	4
Illnesses-US	20	16.1	144	91	144	21	34	17
Illnesses-G	20	12.3	86	67	86	16	26	16
Sodas	28	16.3	108	53	91	15	20	10
Holiday1	24	13	62	54	57	14	19	9
Holiday2	23	17.8	90	44	76	12	17	9
Living Room	33	12.9	107	48	81	10	23	14
Good Leader	36	6.6	151	62	98	25	41	134
GoodTLeader	31	9.7	141	59	98	23	41	29
Good Team1	36	6.4	136	47	87	23	36	46
Good Team3	31	9	135	58	93	21	42	32
Good Team2 Player	36	5.8	136	41	81	24	45	49
BadWords	92	15.8	273	68	113	14	21	10
Industries1	27	34.5	413	184	319	30	46	29
Industries2	43	34.9	510	166	281	29	44	37
CultInd1	44	12.2	299	106	163	26	47	55
CultInd2	29	9.7	203	106	175	29	49	40
Scary Things	99	16.9	453	102	153	18	34	47
MomsOL	55	17.8	389	144	221	20	29	35
MomsF2F	50	30.2	560	193	279	17	25	20
MomsPP	53	16.6	337	115	191	21	33	30
EthnicOL	56	24.6	304	131	189	18	24	15
EthnicF2F	48	33.3	339	130	211	11	21	12
EthnicPP	53	17.9	228	80	137	12	20	11

Increasing the sample size proportionately increases the amount of information, but not always. For Scary Things, 5.6 bits more information were collected per person with full listing (16.9 average list length) than with three or fewer responses per person (3.0 list length); and the number of items obtained in a sample size of 10 with full listing (102) was roughly 5.6 times greater than that obtained with three responses per person (18 items). However, at a sample size of 20 the number of unique items with free lists was only 4.5 times larger (153) than the number obtained with three responses per person (34). *Across examples*, *interviews that obtained more information per person were more productive and obtained more unique items overall even with smaller sample sizes than did interviews with only three responses per person*.

**Fig 2:**
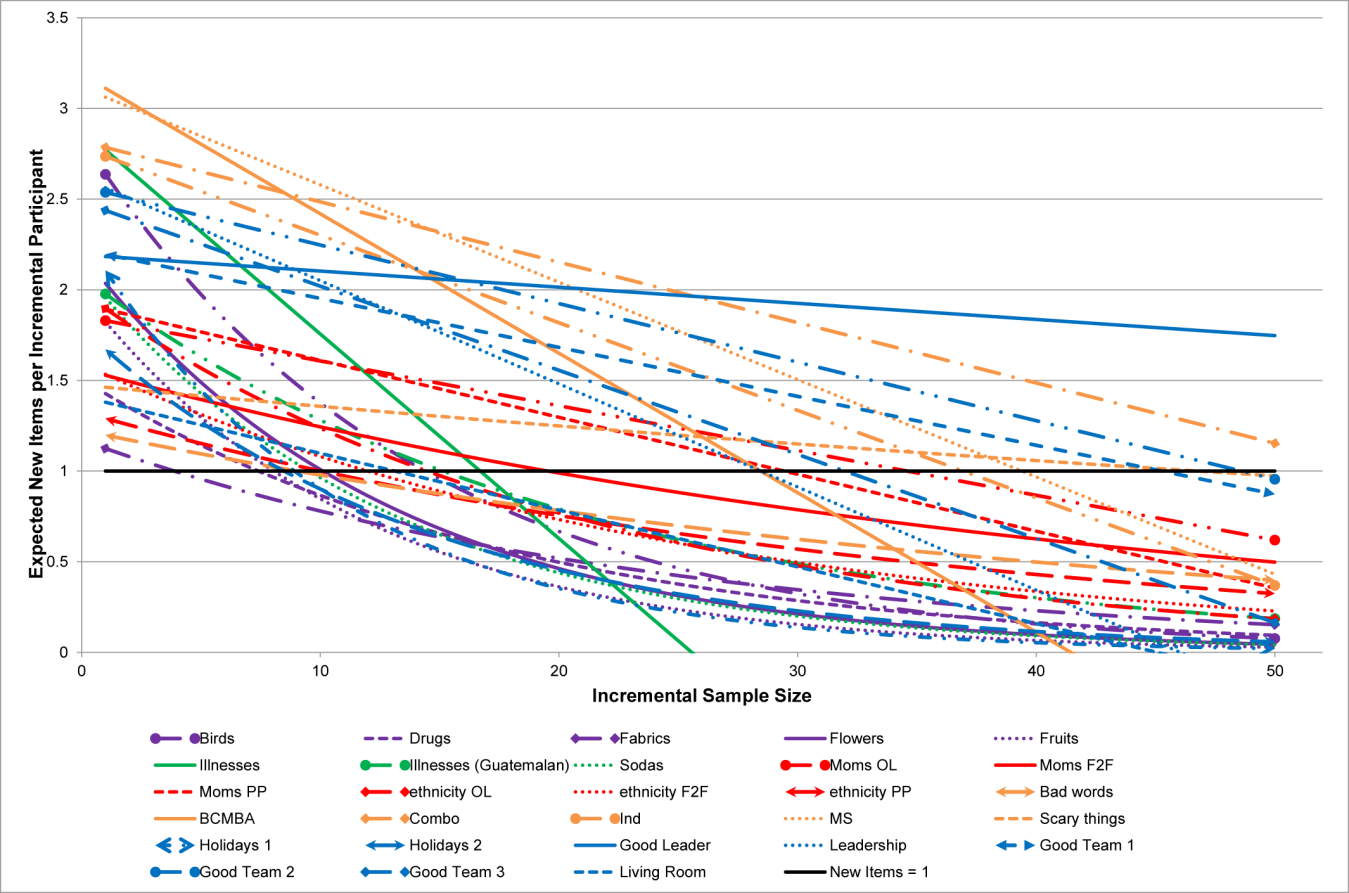
The number of unique items provided with increasing sample size when there are three or fewer responses per person.

Using the same definition of saturation (the point where less than one new item would be expected for each additional person interviewed), less information per person resulted in reaching saturation at much *smaller* sample sizes. Fig 2 shows the predicted curves for all examples when the number of responses per person is three (or fewer). The Holiday examples reached saturation (fewer than one new item per person) with a sample size of 17 (Holiday1) with 13.0 average responses per person and 87 (Holiday2) with 17.8 average responses (Table 2), but reached saturation with a sample size of only 9 (Holiday 1 and Holiday2) when there were a maximum of three responses per person (Table 3, last column). With three or fewer responses per person, the median sample size for reaching saturation was 16 (range: 4–134). Thus, fewer responses per person resulted in reaching saturation at smaller sample sizes and resulted in fewer domain items.

### Salience and sample size

Saturation did not seem to be a useful guide for determining a sample size stopping point, because it was sensitive both to domain size and the number of responses per person. Since a main goal of open-ended interviews is to obtain the most important ideas and themes, it seemed reasonable to consider item salience as an alternative guide to assist with determining sample size adequacy. Here, the question would be: Whether or not complete saturation is achieved, are the most salient ideas and themes captured in small samples?

A simple and direct measure of item salience is the proportion of people in a sample that mentioned an item [37]. However, we examined the correlation between the sample proportions and two salience indices that combine the proportion of people mentioning an item with the item’s list position [13–15]. Because the item frequency distributions have long tails—there are many items mentioned by only one or two people—we focused on only those items mentioned by two or more people (24–204 items) and used the full lists provided by each respondent. The average Spearman correlation between the Smith and Sutrop indices in the 28 examples was 0.95 (average Pearson correlation 0.96, 95%CI: 0.92, 0.98), between the Smith index and the sample proportions was 0.89 (average Pearson 0.96, 95%CI: 0.915, 0.982), and between the Sutrop index and the sample proportions was 0.86 (average Pearson 0.88 95%CI: 0.753, 0.943). Thus, the three measures were highly correlated in 28 examples that varied in content, number of items, and sample size—validating the measurement of a single construct.

To test whether the most salient ideas and themes were captured in smaller samples or with limited probing, we used the sample proportions to estimate item salience and compared the set of most salient items across sample sizes and across more and less probing. Specifically, we defined a set of salient items for each example as those mentioned by 20% or more in the sample of size 20 (because all examples had at least 20) with full-listing (because domains were more detailed). We compared the set of salient items with the set of items obtained at smaller sample sizes and with fewer responses per person.

The set size for salient items (prevalence ≥ 20%) was not related to overall domain size, but was an independent characteristic of each domain and whether there were core or prototypical items with higher salience. Most domains had about two dozen items mentioned by 20% or more of the original listing sample (*n* = 20), but some domains had only a half dozen or fewer items (GoodLeader, GoodTeam2Player, GoodTeam3). With full listing, 26 of 28 examples captured more than 95% of the salient ideas in the first 10 interviews: 18 examples captured 100%, eight examples captured 95–99%, one example captured 91%, and one captured 80% (Table 4). With a maximum of three responses per person, about two-thirds of the salient items (68%) were captured with 20 interviews and about half of the items (53%) were captured in the first 10 interviews. With a sample size of 20, a greater number of responses per person resulted in approximately 50% more items than with three responses per person. Extensive probing resulted in a greater capture of salient items even with smaller sample sizes.

**Table 4:** Capture of salient items with full free list and with three or fewer responses.

	# Free-List Salient Items (≥ 20%, n = 20)	Free-list	Three or Fewer Responses		
		*n* = 10	*n* = 20	*n* = 15	*n* = 10
Fruits	38	100%	36.80%	36.80%	36.80%
Birds	46	100%	50.00%	47.80%	37.00%
Flowers	31	100%	54.80%	48.40%	41.90%
Drugs	21	100%	61.90%	42.90%	42.90%
Fabrics	26	100%	53.8%	53.8%	42.3%
Illnesses-US	22	95.50%	81.8%	77.3%	54.5%
Illnesses-G	21	100%	52.4%	47.6%	47.6%
Sodas	23	100%	69.6%	65.2%	56.5%
Holiday1	20	100%	65.0%	60.0%	50.0%
Holiday2	22	90.90%	72.7%	68.2%	54.5%
LivingRoom	19	100%	73.7%	57.9%	47.4%
GoodLeader	4	100%	100%	100%	100%
GoodTLeader	8	100%	87.5%	75.0%	62.5%
GoodTeam1	6	100%	100%	100%	83.3%
GoodTeam3	6	100%	83.3%	66.7%	66.7%
GoodTeam2 Player	4	100%	100%	100%	100%
BadWords	25	100%	56.0%	56.0%	44.0%
Industries1	48	97.90%	50.0%	47.9%	37.5%
Industries2	49	98.00%	53.1%	49.0%	38.8%
CultInd1	23	95.70%	87.0%	73.9%	65.2%
CultInd2	5	80.00%	100%	80.0%	60.0%
ScaryThings	11	100%	81.8%	72.7%	63.6%
MomsOL	20	95.00%	80.0%	70.0%	65.0%
MomsF2F	31	96.80%	51.6%	51.6%	41.9%
MomsPP	18	100%	83.3%	72.2%	61.1%
EthnicOL	37	97.30%	51.4%	43.2%	37.8%
EthnicF2F	52	100%	30.8%	28.8%	19.2%
EthnicPP	40	97.50%	35.0%	32.5%	20.0%

### Summary and discussion

The strict notion of complete saturation as the point where few or no new ideas are observed is not a useful concept to guide sample size decisions, because it is sensitive to domain size and the amount of information contributed by each respondent. Larger sample sizes are necessary to reach saturation for large domains and it is difficult to know, when starting a study, just how large the domain or set of ideas will be. Also, when respondents only provide a few responses or codes per person, saturation may be reached quickly. So, if complete thematic saturation is observed, it is difficult to know whether the domain is small or whether the interviewer did only minimal probing.

Rather than attempting to reach complete saturation with an incremental sampling plan, a more productive focus might be on gaining more depth with probing and seeking the most salient ideas. Rarely do we need *all* the ideas and themes, rather we tend to be looking for important or salient ideas. A greater number of responses per person resulted in the capture of a greater number of salient items. With exhaustive listing, the first 10 interviews obtained 95% of the salient ideas (defined here as item prevalence of 0.20 or more), while only 53% of those ideas were obtained in 10 interviews with three or fewer responses per person.

We used a simple statistical model to predict the number of new items added by each additional person and found that complete saturation was not a helpful concept for free-lists, as the median sample size was 75 to get fewer than one new idea per person. It is important to note that we assumed that interviews were in a random order or were in the order that the interviews were conducted and were not reordered to any kind of optimum. The reordering of respondents to maximally fit a saturation curve may make it appear that saturation has been reached at a smaller sample size [31].

Most of the examples examined in this study needed sample sizes larger than most qualitative researchers use to reach saturation. Mason’s [6] review of 298 PhD dissertations in the United Kingdom, all based on qualitative data, found a mean sample size of 27 (range 1–95). Here, few of the examples reached saturation with less than four dozen interviews. Even with large sample sizes, some domains may continue to add new items. For very large domains, an incremental sampling strategy may lead to dozens and dozens of interviews and still not reach complete saturation. The problem is that most domains have very long tails in the distribution of observed items, with many items mentioned by only one or two people. A more liberal definition of complete saturation (allowing up to two new items per person) allowed for saturation to occur at smaller sample sizes, but saturation still did not occur until a median sample size of 50.

In the examples we studied, most domains were large and domain size affected when saturation occurred. Unfortunately, there did not seem to be a good or simple way at the outset to tell if a domain would be large or small. Most domains were much larger than expected, even on simple topics. Domain size varied by substantive content, sample, and degree of heterogeneity in salience. Domain size and saturation were sample dependent, as the holiday examples showed. Also, domain size estimates did not mean that there are only 73 fruits, rather the pattern of naming fruits—for this particular sample—indicated a set size of 73.

It was impossible to know, when starting, if a topic or domain was small and would require 15 interviews to reach saturation or if the domain was large and would require more than 100 interviews to reach saturation. Although eight of the examples had sample sizes of 50–99, sample sizes in qualitative studies are rarely that large. Estimates of domain size were even larger when models incorporated item heterogeneity (salience). The Fruit example had an estimated domain size of 53 without item heterogeneity, but 73 with item heterogeneity. The estimated size of the Fabric domain increased from 210 to 753 when item heterogeneity was included.

The number of responses per person affected both saturation and the number of obtained items. A greater number of responses per person resulted in a greater yield of domain items. The bits of information obtained in a sample can be approximated by the product of the average number of responses per person (list length) and the number of people in a sample. However, doubling the sample size did not necessarily double the unique items obtained because of item salience and sampling variability. When only a few items are obtained from each person, only the most salient items tend to be provided by each person and fewer items are obtained overall.

Brewer [29] explored the effect of probing or prompting on interview yield. Brewer examined the use of a few simple prompts: simply asking for more responses, providing alphabetical cues, or repeating the last response(s) and asking again for more information. Semantic cueing, repeating prior responses and asking for more information, increased the yield by approximately 50%. The results here indicated a similar pattern. When more information was elicited *per person*, about 50% more domain items were retrieved than when people provided a maximum of three responses.

Interviewing to obtain multiple responses also affects saturation. With few responses per person, complete saturation was reached rapidly. Without extensive interview probing, investigators may reach saturation quickly and assume they have a sample sufficient to retrieve most of the domain items. Unfortunately, different degrees of salience among items may cause strong effects for respondents to repeat similar ideas—the most salient ideas—without elaborating on less salient or less prevalent ideas, resulting in a set of only the ideas with the very highest salience. *If an investigator wishes to obtain most of the ideas that are relevant in a domain*, *a small sample with extensive probing (listing) will prove much more productive than a large sample with casual or no probing*.

Recently, Galvin [21] and Fugard and Potts [22] framed sample size estimation for qualitative interviewing in terms of binomial probabilities. However, results for the 28 examples with multiple responses per person suggest that this may not be appropriate because of the interdependencies among items due to salience. The capture–recapture analysis indicated that none of the 28 examples fit the binomial distribution. Framing the sample size problem in terms that a specific idea or theme will or will not appear in a set of interviews may facilitate thinking about sample size, but such estimates may be misleading.

If a binomial distribution is assumed, sample size can be estimated from the prevalence of an idea in the population, from how confident you want to be in obtaining these ideas, and from how many times you would like these ideas to minimally appear across participants in your interviews. A binomial estimate assumes independence (no difference in salience across items) and predicts that if an idea or theme actually occurs in 20% of the population, there is a 90% or higher likelihood of obtaining those themes *at least once* in 11 interviews and a 95% likelihood in 14 interviews. In contrast, our results indicated that the heterogeneity in salience across items causes these estimates to underestimate the necessary sample size as items with ≥20% prevalence were captured in 10 interviews in only 64% of the samples with full listing and in only 4% (one) of samples with three or fewer responses.

Lowe et al. [25] also found that items were not independent and that binomial estimates significantly underestimated sample size. They proposed sample size estimation from the desired proportion of items at a given average prevalence. Their formula predicts that 36 interviews would be necessary to capture 90% of items with an average prevalence of 0.20, regardless of degree of heterogeneity in salience, domain size, or amount of information provided per respondent. Although they included a parameter for non-independence, their model does not seem to be accurate for cases with limited responses or for large domains.

## Conclusions

*In general*, *probing and prompting during an interview seems to matter more than the number of interviews*. Thematic saturation may be an illusion and may result from a failure to use in-depth probing during the interview. A small sample (*n* = 10) can collect some of the most salient ideas, but a small sample with extensive probing can collect most of the salient ideas. A larger sample (*n* = 20) is more sensitive and can collect more prevalent and more salient ideas, as well as less prevalent ideas, especially with probing. Some domains, however, may not have items with high prevalence. Several of the domains examined had only a half dozen or fewer items with prevalence of 20% or more. The direct link between salience and population prevalence offers a rationale for sample size and facilitates study planning. If the goal is to get a few widely held ideas, a small sample size will suffice. If the goal is to explore a larger range of ideas, a larger sample size or extensive probing is needed. Sample sizes of one to two dozen interviews should be sufficient with exhaustive probing (listing interviews), especially in a coherent domain. Empirically observed stabilization of item salience may indicate an adequate sample size.

A next step would be to test whether these conclusions and recommendations hold for other types of open-ended questions, such as narratives, life histories, and open-ended questions in large surveys. Open-ended survey questions are inefficient and result in thin or sparse data with few responses per person because of a lack of prompting. Tran et al. [24] reported item prevalence of 0.025 in answers in a large Internet survey suggesting few responses per person. In contrast, we used an item prevalence of 0.20 and higher to identify the most salient items in each domain and the highest prevalence in each domain ranged from 0.30 to 0.80 (Table 1). Inefficiency in open-ended survey questions is likely due to the dual purpose of the questions: They try to define the range of possible answers *and* get the respondent’s answer. A better approach might be to precede survey development with a dozen free-listing interviews to get the range of possible responses and then use that content to design structured survey questions.

Another avenue for investigation is how our findings on *thematic saturation* compare to *theoretical saturation* in grounded theory studies [2,38,39]. Grounded theory studies rely on theoretical sampling–-an iterative procedure in which a single interview is coded for themes; the next respondent is selected to discover new themes and relationships between themes; and so on, until no more relevant themes or inter-relationships are discovered and a theory is built to explain the facts/themes of the case under study. In contrast this study examined thematic saturation, the simple accumulation of ideas and themes, and found that saturation in salience was more attainable–-perhaps more important—than thematic saturation.

## Supporting information

S1 Appendix The original data for the 28 examples. (XLSX)

S2 Appendix GLM statistical model results for the 28 examples. (DOCX)
