# Supplementary material for: Open-ended interview questions and saturation
Source: PLoS One. 2018 Jun 20;13(6):e0198606. doi: 10.1371/journal.pone.0198606 (PMC6010234; doi:10.1371/journal.pone.0198606)
Supplement: S2 Appendix — (DOCX) [file pone.0198606.s002.docx]

**S2 APPENDIX: GLM STATISTICAL MODEL RESULTS FOR THE 28 EXAMPLES**

| **Example** | ***N*** | **Total Items** | **GLM Model**  **Full Free-lists** | **Disp Fac** | **Dist** | **GLM Model**  **Lists with < three responses** | **Dist** |
| --- | --- | --- | --- | --- | --- | --- | --- |
| Fruits | 33 | 62 | exp(2.1654–0.1526X***) | 1.04 | NB | exp(0.6852–0.0851X**) | P |
| Birds | 36 | 121 | exp(2.0745–0.0596X***) | 1.00 | NB | exp(1.0418–0.0723X***) | P |
| Flowers | 41 | 141 | exp(2.3989–0.0744X***) | 0.93 | NB | exp(0.7885–0.0780X***) | P |
| Drugs | 43 | 92 | exp(1.7232–0.0538X**) | 1.08 | NB | exp(0.4112–0.0555X**) | P |
| Fabrics | 63 | 143 | exp(1.3283–0.0178X*) | 1.45 | NB | exp(0.1580–0.0407X**) | P |
| Illnesses-US | 20 | 144 | exp(2.4135–0.0460X) | 0.46 | NB | 2.8842–0.1128X*** | NI |
| Illnesses-G | 20 | 86 | exp(2.4043–0.1161X***) | 0.45 | NB | exp(0.7297–0.0482X) | P |
| Sodas | 28 | 108 | exp(1.9548–0.0469X*) | 0.50 | NB | exp(0.7498–0.0787X**) | P |
| Holiday1 | 24 | 62 | exp(1.9140–0.1127X*) | 2.91 | NB | exp(0.8390–0.0941X**) | P |
| Holiday2 | 23 | 90 | exp(1.5733–0.0182X) | 1.14 | NB | exp(0.5820–0.0686X) | P |
| Living Room | 33 | 107 | exp(1.8418–0.0451X**) | 0.41 | NB | 1.4108–0.0313X | PI |
| Good Leader | 36 | 151 | exp(1.8440–0.0239X*) | 0.13 | NB | 2.1921–0.0089X | NI |
| GoodTLeader | 31 | 141 | exp(1.7801–0.0175X) | 0.24 | NB | 2.6194–0.0569X*** | NI |
| Good Team1 | 36 | 136 | exp(1.6281–0.0170X*) | -- | P | 2.2222–0.0270X* | NI |
| Good Team3 | 31 | 135 | exp(1.7784–0.0203X) | 0.10 | NB | 2.4839–0.0464X** | NI |
| Good Team2 Player | 36 | 136 | exp(1.4698–0.0078X) | -- | P | 2.5698–0.0323X** | NI |
| Bad words | 92 | 273 | exp(1.6576–0.0140X**) | 1.09 | NB | exp(0.2033–0.0225X***) | P |
| Industry1 (BCMBA) | 27 | 413 | exp(3.0172–0.0220X) | 0.61 | NB | 3.1880–0.0769X*** | NI |
| Industries2 (Ind) | 43 | 510 | exp(2.7700–0.0143X) | 0.58 | NB | 2.7841–0.0483X*** | NI |
| CultInd (Combo) | 44 | 299 | 11.6689–0.2158X*** | 0.40 | NBI | 2.8182–0.0333X** | NI |
| CultInd2 (MS) | 29 | 203 | exp(2.6608–0.0543X***) | 0.22 | NB | 3.1158–0.0537X*** | NI |
| Scary Things | 99 | 453 | exp(2.0447–0.0116X***) | 0.45 | NB | exp(0.3883–0.0083X*) | P |
| MomOL | 55 | 389 | exp(2.5654–0.0249X**) | 0.87 | NB | 1.8544–0.0247X** | PI |
| MomF2F | 50 | 560 | exp(2.8326–0.0177X*) | 0.45 | NB | exp(0.4472–0.0229X*) | P |
| MomP&P | 53 | 337 | exp(2.3791–0.0219X**) | 0.54 | NB | 1.9245–0.0314X*** | NI |
| EthOL | 56 | 304 | exp(2.5398–0.0368X***) | 1.32 | NB | exp(0.6872–0.0475X***) | P |
| EthF2F | 48 | 339 | exp(2.5535–0.0282X**) | 1.02 | NB | exp(0.4664–0.0389X**) | P |
| EthP&P | 53 | 228 | exp(2.3873–0.0415X***) | 1.18 | NB | exp(0.2828–0.0282X*) | P |

NI=Normal-identity; NBI=Negative binomial-identity, PI=Poisson-identity; P=Poisson-log; NB=Negative binomial-log

* *p* < 0.05, ** *p* < 0.01, *** *p* < 0.001
